# To the editor

**DOI:** 10.1038/s41525-017-0026-3

**Published:** 2017-06-30

**Authors:** Electron Kebebew, Sudheer Kumar Gara

**Affiliations:** 1 Endocrine Oncology Branch, NCI, NIH, Bethesda, MD USA; 2 George Washington University, Washington, DC USA

We read the article by Gerhard and colleagues with interest.^1^ They report that they have identified a *ZNF23* rs531705739 variant (T40R) by reanalyzing the whole exome sequencing data we shared with them in a kindred we reported on earlier.^2^ We greatly appreciate their effort and interest to reanalyze our data independently. Based on their reanalysis of our whole exome sequencing data, the *ZNF23* rs531705739 variant segregated with affected members in the kindred and was not present in unrelated spouses. They also report a noncoding region that segregates with affected members but do not specify the sequence.

**Fig. 1:**
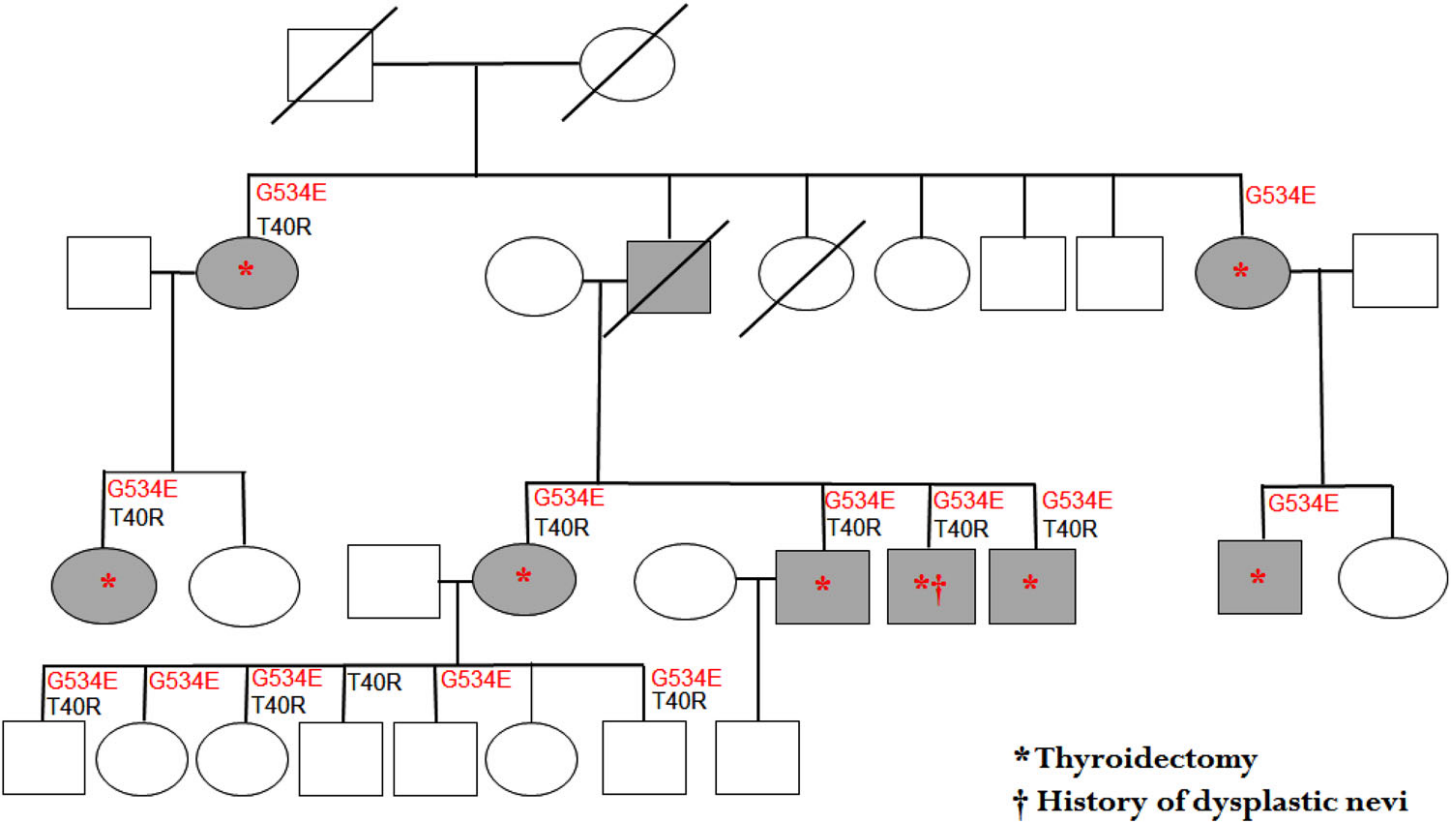
The family pedigree showing the status of ZNF23_T40R and HABP2_G534E variant with respect to non-medullary thyroid cancer. *Squares* denote male family members, *circles* female members, *shaded symbols* affected members and *slashes* deceased members

We performed Sanger sequencing of peripheral blood DNA from the kindred to experimentally validate the findings of Gerhard et al. Although we could validate the *ZNF23* rs531705739 variant (T40R) segregates with six affected family members, two additional family members who developed thyroid cancer during surveillance do not have the variant (Fig. 1). Although, several groups have not validated complete segregation of the *HABP2* (G434E) variant in affected members with familial non-medullary thyroid cancer, the *HABP2* rs7080536 variant (G434E) completely segregates in all the affected members in the kindred including the two newly diagnosed members during surveillance (Fig. 1). We appreciate the efforts of colleagues to independently validate our data as this is the only way we will be able to make progress in identifying true susceptibility gene(s) that cause familial nonmedullary thyroid cancer.
